# Online monitoring of phytate content in plant residuals during wet-treatment

**DOI:** 10.1038/s41598-023-49950-0

**Published:** 2024-01-05

**Authors:** Niklas Widderich, Paul Bubenheim, Andreas Liese

**Affiliations:** Institute of Technical Biocatalysis, Hamburg University of Technology, Hamburg, Germany

**Keywords:** Analytical chemistry, Plant biotechnology

## Abstract

The occurrence of organically bound phosphorus (P) as phytate in plant-based feeding material is a challenge for livestock farming due to limited utilization during the digestion by the animal. Its excretion into the environment through the manure pathway, poses a challenge, due to increased eutrophication and restrictions for P. Hence, while the routine supplementation of phytase enzymes in monogastric diets is common practice, metabolically triggering endogenous plant enzymes by wet-treatment prior to feeding can also lead to a better utilization of phytate bound P and increased digestibility by the animal. Nonetheless, traditional quantification of residual phytate content in plant material is both labor- and chemical-intense. The aim of this study is, therefore, to predict the remaining phytate content during wet-treatment through a straightforward and flexible methodological approach based on real-time analysis. For this, rye bran is used as a model substrate. A partial least squares regression algorithm relates the infrared spectra to the concentrations and predict the amount of P species that are transferred from the bran matrix to the liquid phase. By applying a mass balance for P and considering the effect of water compression, the amount of residual phytate content in rye bran at different time points of wet-treatment is determined. Results are compared to wet chemical methods, resulting in a *RMSEP* of 0.28 g_phytate_∙100 g_bran_^−1^. In addition, the study demonstrates the feasibility of this approach and provides insights into phytate degradation in plant residuals. The method holds the potential for further applications for the screening and investigation of feed material conditioning and also offers the possibility to employ various real-time analytical techniques for assessing phytate remnants in biological samples during wet-treatment.

## Introduction

In livestock nutrition phosphorus (P) is an essential nutrient but often limited in availability^1^. P is stored by the plant in organically bound form as phytic acid (inositol-1,2,3,4,5,6-hexakisphosphate, InsP_6_) or its corresponding salt phytate in a condensed form^2^. It consists of an inositol ring linked to six phosphate groups by ester bonds and accounts for up to 90% of the total P content in most cereals^3^. However, monogastric species, such as swine and poultry, can only poorly digest InsP_6_ present in plant-based feeds due to the limited activity of endogenous intestinal enzymes able to hydrolyze InsP_6_^4^. As a result, almost all dietary InsP_6_ is found in the manure and diluted into the environment, causing P pollution^5^. Besides low P accessibility, the polyanionic InsP_6_ molecule binds multivalent cations (e.g. Ca^2+^, Mg^2+^, Fe^2+^, Zn^2+^), resulting in complexes formed, reducing the bioavailability of these minerals and the P^6^. Furthermore, an impairment of the utilization of other nutrients, such as proteins, starch, and fats, from the diet is also under discussion^7^. Thus, InsP_6_ is considered one of the most important antinutrients in animal feed^6^.

Methods to improve mineral and nutrient digestibility are targeted in animal nutrition, such as the use of various feed treatment processes or the application of exogenous enzymes e.g. phytases, cellulases, xylanases, and amylases^7–9^. Phytases are hydrolytic enzymes that are capable of initiating the stepwise dephosphorylation of InsP_6_, resulting in higher P digestibility and mineral availability^10^. Hence, phytases are important industrial enzymes often supplemented in monogastric animal diets and have the largest market share in the feed industry in the field of enzymes^11^. Nevertheless, due to unfavorable conditions in the intestinal tract, only up to 50% of the InsP_6_ is digested^10^. Besides the application of exogenous enzymes, metabolically triggering the germination process by wet-treatment can effectively reduce the phytate content in feed material^1^. The effectiveness of phytate degradation is a species-dependent phenomenon, which is connected to endogenous phytase activity and is comparatively high in rye bran^1^. It was shown that activation of intrinsic enzymes can completely eliminate the phytate content in rye bran^12^.

For each individual process development (e.g. feed ingredient processing), appropriate analytics to detect the target molecules are crucial. Since the beginning of the nineteenth century, much effort has been made to detect and quantify InsP_6_ present in plant material^13^. However, most of the methods developed are based on protracted extraction procedures and quantification by chromatography. Limitations are often the large sample volumes required and tedious preparation steps that render these methods slow, expensive, and sometimes environmentally unfriendly^14^. Although offline analytical methods allow quantification of the target compound, it is often not possible to react appropriately to changing process parameters and fluctuations during the ongoing process. Thus, a real-time analytical method is advantageous, especially, when the starting material is of biological origin and, therefore, subject to natural variations^15^.

Fourier-Transform-Infrared (FT-IR) spectroscopy is an analytical technique based on the excitation of energy states in molecules. Infrared radiation interacts with specific vibrations of molecular bonds. As a result, the emitted infrared radiation is absorbed and molecular-specific absorption spectra are obtained. The advantages are the minimal sample preparation required and the ability to utilize the same protocol for any plant tissue^14^. Infrared light in the mid-infrared region (MIR, 4000–400 cm^−1^) of the electromagnetic spectrum is used extensively in the determination of plant constituents; e.g. to determine compositions of lignocellulose materials, quality of milling fractions in wheat and compositional changes during seed germination^16–18^.

FT-MIR is also used for the identification and quantification of many different inorganic phosphates; as well in biological matrices such as char^19,20^. According to Campos et al. who studied inorganic phosphates from natural, weathered, and biogenic origin, characteristic bands for phosphate ion (PO_4_^3−^) appear in the range of 1182–1005 cm^−1^ and 634–450 cm^−1^, corresponding to asymmetric stretching of P–O vibrations and bending deformation of O–P–O, respectively^21^. In another study by Grunenwald et al.^22^ it is proposed to use the PO_4_ absorbance in the range of 1230–900 cm^−1^ to determine the carbonate content of apatite from the ratio of PO_4_–CO_3_. However, all investigations were done using solid and dry material. Greater challenges arise when measuring in aqueous systems, such as during wet-treatment of plant materials, as large parts of the IR-spectrum are covered by OH–bands. Attenuated total reflection Fourier-Transform-Infrared (ATR-FT-IR) spectroscopy is a highly versatile, label-free, and non-invasive imaging method. The major advantage of ATR-FT-IR is the opportunity to measure samples that absorb strongly in the IR-spectrum^23^. Morisset et al. used ATR-FT-MIR to quantify orthophosphate in aqueous solution during microalgae cultivation. A partial least squares regression (PLSR) algorithm relates the spectral intensities to the concentrations. The model was calibrated with KH_2_PO_4_ in the range of 1771–1001 cm^−1^^24^.

Initial results have also been published in InsP_6_-analysis. He et al.^25^ characterized different metal phytate compounds and suggested that some spectral features of metal phytates could be used to distinguish phytate compounds from metal phosphate compounds. Guan et al. investigated the complexation behavior of phytate with aluminum hydroxide using FT-MIR. The results revealed that three out of six phosphate groups bound to aluminum hydroxide^26^. Recently, a method for rapid nutritional profiling of pea seeds was developed^14^. However, we show here that the absorption bands of orthophosphate and phytate are almost identical in aqueous solution, which makes discrimination by FT-MIR challenging. Nevertheless, a real-time analytical approach utilizing ATR-FT-MIR and allowing the quantification of residual phytate content in plant material during wet-treatment was developed. Notably, the water absorption by the plant material is taken into account. The approach is evaluated for its feasibility and applicability and used to better understand phytate degradation during wet-treatment.

## Material and methods

Unless otherwise stated, all chemicals used were of analytical grade or higher quality. Rye bran was provided by *Aurora Mühlen GmbH* (Hamburg, Germany) containing a common mixture from different cultivation areas in Germany.

### Phosphorus content and intrinsic phytase activity

A method of the Association of Official Analytical Collaboration (AOAC) was used to determine the total phosphorus content in rye bran^28^. Samples of 1 g plant material were thoroughly dried at 105 °C for at least 4 h. Dried samples were incinerated at 550 °C and extracted in 10 ml of 6 M HCl, heated to the boiling point.

Dissolved inorganic P content was analyzed using a molybdenum blue color developing reagent. The color-developing reagent consisted of four parts acidic 0.012 M ammonium heptamolybdate tetrahydrate (Carl Roth GmbH & Co. KG, Karlsruhe, Germany) solution and one part of 0.711 M FeSO_4_·7 H_2_O (Sigma-Aldrich, Steinheim, Germany) solution. After 5 min incubation, the absorbance of the color complex formed was measured at 700 nm (Shimadzu UV-1280). For quantification of free inorganic P, a standard curve of KH_2_PO_4_ (Carl Roth GmbH & Co. KG, Karlsruhe, Germany) was used.

The determination of intrinsic phytase activity follows a modified protocol of Eeckhout and DePeape^29^. 0.2 g of rye bran was incubated in 50 ml reaction solution at 37 °C and stirred at 120 rpm (SW 23, Julabo Labortechnik GmbH, Seelbach, Germany). The reaction solution was a 0.0015 M phytic acid solution (Sigma-Aldrich, Steinheim, Germany) of 180 ml H_2_O and 820 ml of a 0.25 M potassium acetate (Carl Roth GmbH & Co. KG, Karlsruhe, Germany) buffer. The pH was adjusted to 5.5 with H_2_SO_4_. After 10 min and 70 min 50 µl sample were taken and immediately mixed with 450 µl of 0.612 M trichloroacetic acid (Carl Roth GmbH & Co. KG, Karlsruhe, Germany) and 500 µl of the molybdenum blue color developing reagent. The difference in liberated inorganic phosphorus after 10 min and 70 min was used to determine the activity (expressed in µmol min^−1^ per kg of biological material as dry matter). Thereby, the phytase unit is defined as the amount of inorganic P released in one minute from a 0.0015 M phytate solution at pH 5.5 and 37 °C.

### Phytate extraction and quantification

To determine the initial and the residual amount of phytate present in rye bran, an HPLC method from Sandberg et al. was used in a modified manner^30^. Prior to analysis, plant material was resuspended in 0.5 M HCl at a ratio of 1:7 (w/v) and incubated for 16 h (at 37 °C, stirred at 250 rpm) for phytate extraction. For purification, a modified protocol from Herrmann et al.^3^ was used. In brief summary, HCl extracts were diluted 1:5 in H_2_O and applied to HyperSep™ SAX cartridges (Thermo Fisher Scientific, Darmstadt, Germany) using reduced pressure (800 mbar). Samples were eluted with 2 M HCl, evaporated (Rotavapor R-200, Büchi Labortechnik AG, Flawil, Switzerland), and phytate was dissolved in 50 mM sodium acetate (Carl Roth GmbH & Co. KG, Karlsruhe, Germany) pH 5 for HPLC analysis. A reversed-phase RP18 analytical column (LiChrospher®100, 5 µm, 250 mm x 4 mm) was used with a mobile phase consisting of MeOH (Carl Roth GmbH & Co. KG, Karlsruhe, Germany), H_2_O, formic acid (Carl Roth GmbH & Co. KG, Karlsruhe, Germany), and tetrabutylammonium hydroxide (40% in H_2_O, Tokio Chemical Industry, Tokyo, Japan). Inositol phosphates were detected at 39 °C using a refractive index detector. Typical retention times were 8 min (InsP_3_), 12 min (InsP_4_), 16 min (InsP_5_), and 23 min (InsP_6_).

For comparability and appropriate presentation, the phytate content is expressed in inositol-phosphorus (inositol-P) content, given as gram P bound to inositol-P per 100 g of bran. Inositol-P refers to the P atoms attached to the inositol phosphates and is derived from the molar mass of phosphorus. This results in conversion factors of 0.14, 0.19, 0.23, and 0.28 for InsP_3_, InsP_4_, InsP_5,_ and InsP_6_, respectively.

### Water uptake

Particle size distributions were determined in the interval 0.1–2000 µm, in triplicates. The cumulative and volume-specific particle size distribution Q_3,dry_ of the dry plant material was determined by imaging (Camsizer XT, Microteac Retsch GmbH, Haan, Germany), allowing to derive volume-specific surface area S_V_ (µm^2^ µm^−3^) and sphericity Ψ. Q_3,soaked_ of the soaked plant material was measured in suspension by laser diffraction (LS 13 320, Beckman Coulter Life Sciences, Brea, USA) applying the Frauenhofer optical model. The density of the bran bulk was determined by means of gas displacement measurement (AccuPyK II, Micrometrics Instrument Corporation, Norcross, USA).

Water uptake of the bran bulk was mathematically derived from the dry and soaked particle size distributions. The Factor *F*_*swell*_, Eq. (1), on average reflects the volume increase of the bran bulk after soaking. The sphericity *Ψ* and the volume-specific surface area *S*_*V*_ are used to calculate the Sauter mean diameter *d*_*2,3 dry*_ (µm), Eq. (2), of dry particles. By applying the Sauter mean diameter the mean volume of dry particles *V*_*2,3 dry*_, Eq. (3), was determined. The volume ratio of dry to soaked particles is described by *F*_*swell*_ and thus *V*_*2,3 soaked*_ could be derived, Eq. (4). The difference between *V*_*2,3 soaked*_ and *V*_*2,3 dry*_ corresponds to the average water volume absorbed *V*_*H2O*_, Eq. (4). By correlation of the density of the bulk particles and *V*_*2,3 soaked*_, the amount of water absorbed was derived and expressed in mass-specific terms, given in ml_water_ g_bran_^−1^.

1 $$F_{swell} = \frac{{\smallint Q_{3} \left( x \right)_{dry} }}{{\smallint Q_{3} \left( x \right)_{soaked} }}$$

2 $$d_{2,3 dry} = \frac{6}{{\Psi \cdot S_{V} }}$$

3 $$V_{2,3 dry} = \frac{6}{\pi }d_{2,3 dry}^{3}$$

4 $$V_{2,3 soaked} = \frac{6}{\pi }d_{2,3,dry}^{3} \cdot F$$

5 $$V_{H2O} = V_{2,3 soaked} - V_{2,3 dry}$$

### ATR-FT-MIR spectroscopy

#### Data collection

FT-MIR-ATR measurements were performed using a Vertex 70 (Bruker Optik GmbH Co. KG, Ettlingen, Germany) spectrophotometer equipped with an optical fiber (Fiber Probe IN 350-T, Bruker Optik GmbH Co. KG, Ettlingen, Germany). The optical fiber has an ATR cell implemented, consisting of a diamond prism (2 reflections, 45° angle of incidence) as the internal reflection element. For detection, a thermoelectrically cooled MCT (Mercury-Cadmium-Telluride) detector at a scanner velocity of 40 kHz was used. Spectra were taken at a resolution of 4 cm^−1^ in the wavenumber range of 4000–600 cm^−1^. Each ATR spectrum represents an average of 200 scans and was recorded with the blank ATR configuration as the background. To minimize the interference from water vapor and carbon dioxide absorption bands, the device was purged with dry nitrogen. The ATR cell was cleaned after each experiment by washing it with ultrapure water and ethanol until no significant bands due to impurities appeared in the blank ATR configuration. In this study the part of the mid-infrared region (1350–1020 cm^−1^) was investigated, which covers the bands associated with various P–O(H) and P=O vibrations. Measurements were always done in the aqueous phase.

#### Data analysis

Partial least square algorithm (PLSR) was used for quantitative analysis; i.e., to predict the amount of inorganic P in the liquid phase. This method with a calibration series of KH_2_PO_4_ (Carl Roth GmbH & Co. KG, Karlsruhe, Germany) at 18 different concentrations ranging from 0 to 200 mM were used to develop a calibration model. To increase the model´s accuracy, a spectrum of each concentration was taken twice and the duplicates were averaged for calibration. The pH was adjusted to 6.5 with HCl, as this is the naturally occurring pH of the bran-water suspension at the given bran to water ratio. In addition, KH_2_PO_4_ was used for the determination of the probe response time and the detection limit.

The spectra were cut to the fingerprint region from 1700 to 900 cm^−1^ covering the target molecular vibrations. In addition, the data preprocessing techniques included a baseline offset and a maximum normalization at the water band appearing at 1600 cm^−1^ using *The* *Unscrambler X* statistical software (version 10.5.1, Camo Software, Oslo, Norway). Based on this, the PLSR model was trained in the range of 1350–1020 cm^−1^.

The number of factors for the PLSR model was selected for an optimum between error of calibration and error of prediction. Accordingly, the lowest possible root mean square error of cross validation (RMSECV), Eq. (6), was aimed for without overfitting the model. A full cross validation was performed, i.e. each averaged spectrum was omitted and predicted once. Furthermore, the prediction accuracy of the final model to predict the total P content in the liquid phase is given by the root mean square error of prediction (RMSEP), Eq. (6). For calculation, the total P concentration in the liquid phase after 1 h, 2 h, 4 h, 8 h and 16 h of wet-treatment was determined colorimetrically by molybdenum blue color reaction. For additional evaluation of the model´s performance, the coefficient of determination *R*^*2*^ was determined. An *RMSE(-P/-CV)* close to zero and a *R*^*2*^ close to one shows a good agreement with the data.

6 $$RMSE\left( { - P/ - CV} \right) = \sqrt {\frac{{\mathop \sum \nolimits_{i = 1}^{n} (y_{i} - \hat{y}_{i} )^{2} }}{n}}$$

*RMSEP* root mean square error of prediction (mM or g_P_∙100 g_bran_^−1^), *RMSECV* Root Mean Square Error of Cross Validation (mM or g_P_∙100 g_bran_^−1^), *y*_*i*_ concentration determined by the reference method (mM or g_P_∙100 g_bran_^−1^), *ŷ*: concentration determined by the PLSR model using MIR data (mM or g_P_∙100 g_bran_^−1^), *n* total number of samples

### Prediction of residual phytate content during wet-treatment

Since the training data set only maps the P–OH and P=O vibration patterns in the liquid phase, the phytate concentration in rye bran during wet-treatment was determined via a mass balance, Eq. (7). It was assumed that all P–OH and P=O absorption patterns in the liquid phase are either attributed to phytic acid or orthophosphate (PO_4_^3−^, hydrolysis product). Due to different phosphorylation states of phytic acid (InsP_1_–InsP_6_) in rye bran, the mass balance was normalized to P (31 g mol^−1^) and refers to P bound to inositol phosphate. In addition, the compression of the aqueous phase due to the water absorption of bran particles is included in the mass balance.

7 $$\begin{aligned} \frac{{dm_{P} }}{dt} = 0 = & m_{InsP,bran} \left( {t_{0} } \right) - m_{InsP,bran} \left( t \right) - m_{P,liquid phase} \left( t \right) \\ m_{P,liquid phase} \left( t \right) = & \frac{{c_{measured} \left( t \right) \cdot V\left( {m_{bran} } \right)_{water} \cdot M_{P} }}{{m_{bran} }} \cdot 100 \\ \end{aligned}$$

$m_{InsP,bran} \left( {t_{0} } \right)$: amount of inositol-P present in starting material (g_P_∙100 g_bran_^−1^), $m_{InsP,bran} \left( t \right)$: amount of inositol-P present at time point t (g_P_∙100 g_bran_^−1^), $m_{P,liquid phase} \left( t \right)$: amount of P in liquid phase at time point t (g_P_∙100 g_bran_^−1^).

where *c*_*measured*_*(t)* is the feedback variable. It is the concentration of P–O and P=O valence vibrations in the aqueous phase, which is determined from the MIR data via the PLS model. *V(m)*_*liquid*_ is the volume of the liquid phase. Due to water absorption of the bran particles, it is a factor depending on the amount of bran. Consequently, the actual volume of the liquid phase is lower than the liquid volume introduced to the system at the beginning. To verify the accuracy of the mass balance, the residual phytate content after 1 h, 2 h, 4 h, 8 h, and 16 h of wet-treatment was determined by extraction and HPLC analysis and compared with the mass balance results taking into account the predicted concentration by the PLSR model at the same time points. The result of that comparison is expressed as RSMEP, Eq. (6). In addition, the coefficient of determination *R*^*2*^ is determined. For further verification of the mass balance, the colorimetrically determined P content in the liquid phase (which is also used to calculate the *RMSEP* of the PLSR model) is also used as a feedback for the mass balance, to determine the residual phytate content in rye bran. These results are also compared with the mass balance results using the FT-MIR data. For comparability the results are also expressed as *RMSEP*, Eq. (6), and the coefficient of determination *R*^*2*^ is given.

### Experimental procedure

**Figure 1:**
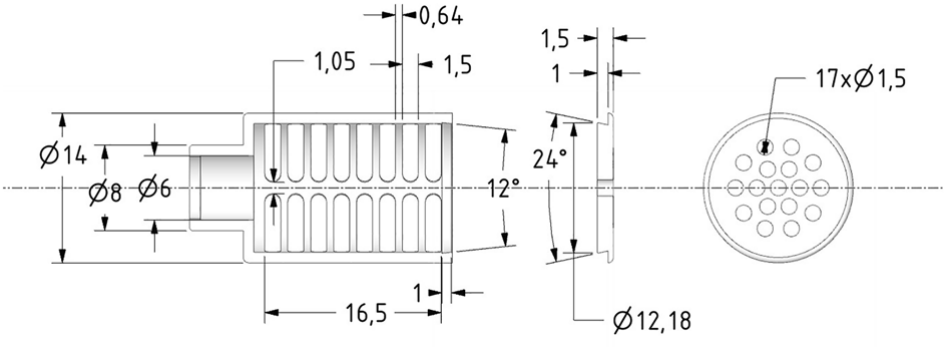
Membrane module to retain bran particles, made out of POM (polyoxymethylene) and covered by a nylon membrane (mesh size < 10 µm, Durapore® Membrane Filter, Merck Millipore, Burlington, USA). Length specifications in mm.

Experiments were carried out in glass beakers at 20% (w/v) bran to H_2_O ratio. No further additives were used. Therefore, the reduction of phytate content during wet-treatment was realized solely by mass transfer (solubilization in H_2_O) and intrinsic enzyme activity. The suspension was stirred with a magnetic stirrer at 300 rpm to ensure homogeneous mixing. In order to prevent particle sedimentation on the ATR-crystal and thus blindness of the optical fiber, a nylon membrane (mesh size < 10 µm, Duropore® Membrane Filter, Merck Millipore, Burlington, USA) was used. The membrane allows convection and diffusion of all substances in solution without interfering with the measurement while retaining the bran particles. To avoid contact of the membrane with the ATR crystal, a module (protective cage) was made of polyoxymethylene (POM), Fig. 1, and is enclosed by the membrane.

After distinct time points (1 h, 2 h, 4 h, 8 h, and 16 h) wet-treatment was stopped by transferring the suspension to centrifuge tubes followed by a repeated washing procedure to remove the P species in solution. The suspensions were three times centrifuged (5000 rpm, 5 min, Universal 320 R, Andreas Hettich GmbH & Co. KG, Tuttlingen, Germany), the supernatant removed, and the bran fraction resuspended in H_2_O. A sample was taken from the supernatant of the first centrifugation and the P-content was measured using the molybdenum blue color reaction. After the final washing step, the residual phytate in the plant material was extracted and quantified via HPLC analysis.

### Ethical approval

All institutional, national, and international guidelines and laws on plant research have been adhered to. The necessary permits and approvals to ensure the ethical treatment of plants and the conservation of biodiversity have been obtained. Notably, this study does not involve any work with threatened species. The grain is commercially supplied by Aurora Mühlen GmbH. All authors have the deepest reverence for nature. Specimens of rye bran were retained at the Institute of Technical Biocatalysis and can be obtained from the corresponding author upon justified request.

## Results and discussion

### Physiochemical properties of rye bran

The analysis of the rye bran showed a total P content of 1.3 g_P_·100 g_bran_^−1^ and an inositol-P content of 0.97 g_P_·100 g_bran_^−1^ (equaling 3.4 g_phytate_∙100 g_bran_^−1^). Compared to literature, these values are slightly elevated^31^ but might be subject to biological variations caused by different growing conditions, e.g. cultivation year^32^ and harvest region^33^. However, the ratio of inositol-P to total-P measured is 0.74 and by this corresponds to descriptions in literature^29,31^. Further analysis showed a distribution of differently phosphorylated inositols in rye bran of 0.56 mol-% InsP_3_, 1.15 mol-% InsP_4_, 6.67 mol-% InsP_5,_ and 91.61 mol-% InsP_6_. Furthermore, the intrinsic phytase activity was determined to be 4955 U kg_bran_^−1^ which is consistent with literature data^31,34^.

**Figure 2:**
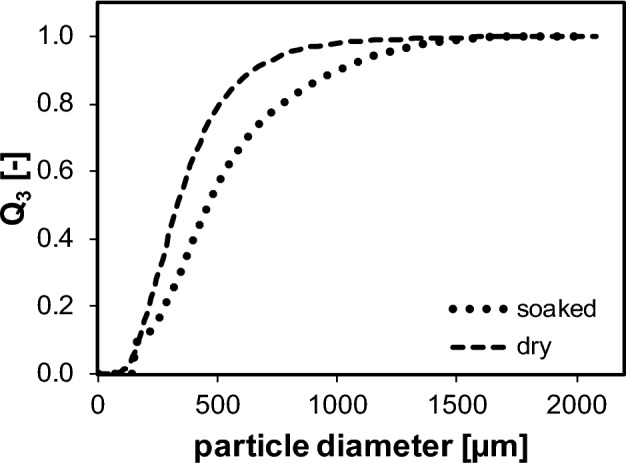
Cumulative and volume-specific particle size distributions of dry and soaked bran particles. Distributions are used to calculate an average swelling factor and to derive particle properties. *Q*_*3,dry*_ is determined by imaging, whereas *Q*_*3,soaked*_ is measured in suspension by laser diffraction applying the Fraunhofer optical model^35^.

No change in particle size distribution could be observed after 15 min of wet-treatment. Due to this, a time-independent value for the water absorption is derived from the average volume increase due to swelling. For this, the volume-specific particle size distributions of the dry and soaked bran bulk, Fig. 2, are used to derive a factor *F*_*swell*_ of 1.164 (Eq. 1). Soaked Particles < 150 µm were neglected due to uncertainties of the measurement, resulting in a deviation in the range of 10^–3^. Taking into account the sphericity *Ψ* and the volume-specific surface area *S*_*V*_ of the dry bran bulk, the Sauter mean diameter *d*_*2,3 dry*_ is 509 µm and the density of the bran bulk *ρ*_*bran*_ is 1.49 g·cm^−3^. These physical properties result in a water absorption of 0.39 ml_water_ g_bran_^−1^ applying Eqs. (1)–(5). This value, therefore, reflects the average water absorption due to the volume increase.

### Comparison of IR absorption behavior of KH_2_PO_4_ and phytic acid

**Figure 3:**
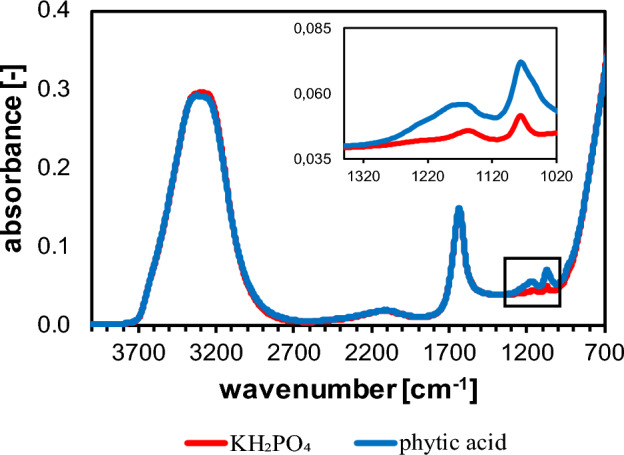
Absorbance pattern of 25 mM KH_2_PO_4_ and 25 mM phytic acid in aqueous solution at room temperature and pH 6.5. The image within the figure shows the calibration range for the PLSR model.

KH_2_PO_4_ is used for quantitative analysis of inorganic P. As shown in Fig. 3, KH_2_PO_4_ and phytic acid show similar MIR-absorption behavior in aqueous solution, with the only difference being, that according to the degree of phosphorylation of phytic acid, the absorbance proportional increases. According to Guan et al.^26^ the two most dominant peaks at 1172 and 1065 cm^−1^ are assigned to the asymmetric and symmetric stretching vibrations of P–O in HPO_3_^−^, respectively, both of which are present in KH_2_PO_4_ (dissociated) and phytic acid. Karunakaran et al.^14^ studied the IR-absorption behavior of phytic acid and further classified the characteristic peak in the range of 1100–1060 cm^−1^ as O=P–O vibration. Nevertheless, this vibration pattern is also found in both substances, making it challenging to distinguish them. Further peak assignments in the region below 1000 cm^−1^ were made by Guan et al.^26^. However, in this study, these peaks are superimposed by water absorption, as is the OH-band at 3500 cm^−1^, and, therefore, are not considered. Both KH_2_PO_4_ and phytic acid show a very similar IR-absorption pattern in aqueous solution. Moreover, KH_2_PO_4_ can be obtained in much higher purity than phytic acid^36^, making it suitable to quantify P–O and P=O species, including phytic acid, in solution.

For quantification of P–O and P=O species in aqueous solution, the frequency range of 1350–1020 cm^−1^ is chosen, as it covers all relevant P–O and P=O vibrations that are not superimposed by OH-bands (Fig. 3). Morisset et al. chose a frequency range of 1771–1001 cm^−1^. The large integration range was set for quantification of several components such as carbonate and bicarbonate, whereas only the absorbance in the range of 1116–1001 cm^−1^ is caused by orthophosphate (HPO_4_^2−^)^24^. In this study, however, the best predictions were obtained for an extended frequency range up to 1350 cm^−1^, as shown in Fig. 3.

### Chemometrics

**Figure 4:**
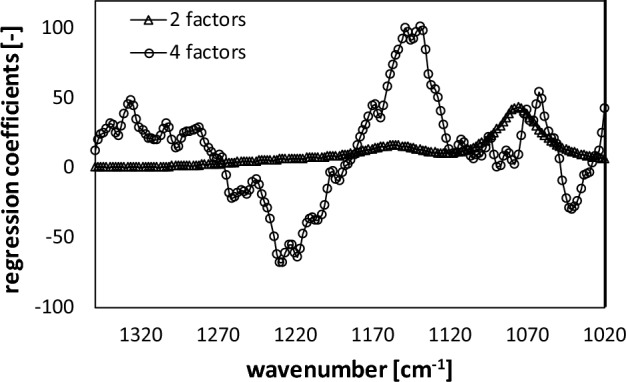
PLSR regression coefficients for two factors (*RMSECV* of 2.15 mM) and four factors (*RMSECV* of 1.05 mM).

Partial least squares regression (PLSR) is a multivariate analysis method that constructs spectral data into factors and develops predictive models using factors when they are highly collinear^14^. In this study, PLSR modeling is used to relate IR-absorption patterns to the concentrations of total P-OH and P=O species in aqueous solution. The training data set with KH_2_PO_4_ experienced a *RMSECV* of 2.15 mM KH_2_PO_4_, equaling 0.36 mM InsP_6_, with a coefficient of determination R^2^ of 0.99. It shows that the calibration data is of high agreement with the model prediction, resulting in reliable results. In addition, best predictions were achieved using two factors that already cover 100% of the spectral variance. However, the lowest *RMSECV* is given by four factors, resulting in an *RMSECV* of 1.08 mM KH_2_PO_4_. Nevertheless, more than two factors lead to overfitting, and noise in the data is described by the PLSR model, resulting in overestimation of concentrations. This behavior is also reflected by the regression coefficients (Fig. 4)^37^.

The emergence of noise in the regression coefficients is manifested by the reduction in the structure and the existence of sharp peaks with a high degree of directional oscillation^38^. Figure 4 shows that when two factors are employed, the course of the regression coefficients has an unambiguous and smooth structure, aligning with the spectra. In contrast, more than two or even four factors lead to the described behavior and noise is included, leading to overfitting and erroneous predictions.

The predicted values are compared to colorimetrically determined inorganic P concentrations in the liquid phase after different times of wet-treatment (1 h, 2 h, 4 h, 8 h, and 16 h), resulting in a *RMSEP* of 6.32 mM KH_2_PO_4_ and the coefficient of determination *R*^*2*^ of 0.87. Thus, the chemometric and colorimetric data have a high degree of agreement. The lower *RMSECV* (2.15 mM) of the calibration data set compared to the *RMSEP* (6.32 mM) shows, that the model is reliable, and also indicates that the model is not over-fitted. Furthermore, the *R*^*2*^ reflects a high model certainty. Thus, the model is suitable for predicting P–OH and P=O species in the aqueous phase of the bran-water suspension. It is also evident from this data set that the phytate contained in bran is already degraded in the plant matrix and confirms the assumption that all P-species passed to the liquid phase can be assigned to orthophosphate.

Based on the PLSR model, the lower limit of quantification is 10 mM KH_2_PO_4_, equaling 1.67 mM InsP_6_, which corresponds to standard RP-18 chromatography. Morisset et al. also determined PO_4_^3−^ (KH_2_PO_4_) concentrations in aqueous solution using ATR-FT-MIR with a similar limit of quantification^24^. Thus, the membrane covering the ATR-probe does not affect the concentration of P species at the ATR-crystal after an equilibrium is reached. Furthermore, the probe response time at a stirrer speed of 300 rpm is 216 s. The acquisition of the 200 spectra takes 56 s and is already included in the response time of the probe. Compared to sampling, sample preparation, and offline quantification, this represents a significant reduction in the usage of chemicals and time. In addition, if hours of wet-treatment are considered, the response time has only a minor influence on the final result. Moreover, the response time can be shortened by increased convection in the system if another setup with a firmly integrated stirrer and rotor, e.g. bioreactor, is used.

### Quantification of residual phytate content

**Figure 5:**
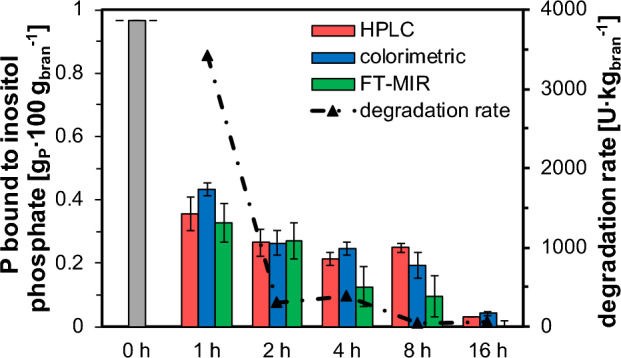
Comparison of chemometric data including the mass balance, Eq. (7), for quantification of residual phytate content after different wet-treatment times with wet chemical analytical methods. Wet chemical analytical methods include colorimetrically determined P-content in the aqueous phase and extraction and HPLC analysis for residual phytate content. The colorimetric data is also used as feedback for the mass balance. Chemometric determined data is used to calculate the degradation rates between the treatment times.

The objective of this study was to predict the residual phytate content in rye bran during wet-treatment using a simple and adaptable methodological approach. Time-dependent P concentrations in the liquid phase resulting from FT-MIR measurements and chemometrics are used as a feedback variable in a mass balance, Eq. (7), to infer the residual phytate content. The results are compared to extraction and HPLC analysis, Fig. (5), resulting in an *RMSEP* of 81 mg_P_ 100 g_bran_^−1^ and a coefficient of determination *R*^*2*^ of 0.78. This corresponds to a deviation of about 8% related to the total inositol-P content present in the biological starting material. Taking into account the molecular weight of phytic acid (660 g mol^−1^), the *RMSEP* related to phytate is 0.28 g_phytate_ 100 g_bran_^−1^. In Fig. 5 it can be seen that the deviation of the data sets is in the range of measurement uncertainties. Small deviations such as the higher residual phytate content after 8 h compared to the measurement after 4 h of wet-treatment determined by HPLC are thermodynamically impossible and can be explained by the biological origin of the substrate, since a new batch was prepared for each experiment. This is also reflected by the apparently low coefficient of determination *R*^*2*^ of 0.78. In contrast, the result of the mass balance using chemometric as well as colorimetric feedback shows the expected trend, also suggesting that the HPLC data for this time point is slightly erroneous.

Comparison of the results from the colorimetric measurements with the chemometric data, both including the mass balance, gives a similar *RMSEP* of 86 mg_P_ 100 g_bran_^−1^ and accordingly 0.30 g_phytate_ 100 g_bran_^−1^. with a coefficient of determination *R*^*2*^ of 0.86, demonstrating the robustness of the developed methodology. Based on all data sets, it is concluded that the mass balance is valid. As a conclusion one can state that the developed method for the prediction of the residual phytate content provides as accurate results as the most commonly used methods for inorganic P and inositol phosphate quantification, and is significantly faster, less laborious, and non-invasive in comparison.

Neglecting water absorption by bran particles, the *RMSEP* is 145 mg_P_∙100 g_bran_^−1^ and accordingly 0.512 g_phytate_∙100 g_bran_^−1^, leading to significant deviations without applicability. Therefore, the consideration of water absorption in the mass balance is of particular importance for the accuracy of the results. Furthermore, the precision of results also confirms the mathematical derivation of the water absorption, which can in principle be applied to various particle distributions of biological origin with similar swelling behavior.

This methodological approach is based on real-time analysis, the residual phytate content is determined during the treatment and not only after treatment, which provides more accurate insights into phytate degradation. In this context, Fig. 5 also shows that phytate degradation is detected up to 16 h of wet-treatment, whereas even after 16 h small amounts of residual phytate (3.8% remaining) are still detected in the biological substrate. Considering the intrinsic enzyme activity and the amount of substrate, a treatment of more than 16 h is recommended for complete degradation of the contained phytate. Reddy et al. showed complete phytate degradation after 5 days of wet-treatment at 25 °C^12^. However, most of the phytate (63%) is initially degraded within the first hour of wet-treatment and the degradation rate steadily decreases between subsequent time points. Phytate degradation is a species-dependent phenomenon and is linked to intrinsic phytase activity^1^. Therefore, different time periods for degradation are required for different biological starting material. However, the wet-treatment with 16 h already represents a significant improvement compared to phytase supplementation to animal feed rations, where 50% of phytate ends up in manure^10^. If other biological matrices with similar intrinsic phytase activity such as wheat^39^ are considered, a similar degradation behavior is expected.

### Phytate degradation pattern during wet-treatment

Besides determination of the residual phytate content, the developed approach is used to illustrate the degradation pattern of phytate in plant material during wet-treatment. It can be seen from Fig. 5 that the degradation of phytate follows neither simple zero-order nor first-order kinetics, since the rate neither remains constant nor decreases proportionally. Therefore, either a limitation of mass transfer or a decrease in enzyme activity during wet-treatment can be concluded. Certainly, a decrease in reaction rate is observed during wet-treatment as the substrate concentration in the biological matrix decreases over time. However, as the decrease in enzyme activity does not follow a linear trend, it is very likely that the degradation is mass transfer limited. Considering 1 h of conditioning the degradation rate is 3263 U kg_bran_^−1^. The conventionally determined intrinsic enzyme activity is 4955 U kg_bran_^−1^, resulting in a deviation of 34%. This deviation might be within biological fluctuations, but may also be due to the different substrate concentrations and process conditions (pH of 6.5 compared to 5.5). However, the conventional approach only considers the release of inorganic P from extrinsic phytate standards, whereas the chemometric analysis also takes the mass transfer of phytate into account. Thus, this rate does not correspond exactly to the intrinsic enzyme activity. However, this analysis provides more valuable information from a process engineering point of view, showing a more realistic behavior of intrinsic phytate degradation and suggest mass transfer limitation during wet-treatment.

## Conclusion

Most research on phytic acid is aimed at dephosphorylating the molecule in plant material to reduce the negative impact of livestock farming on the environment. Commonly employed methods for quantitative analysis of phytic acid are time-consuming and expensive. In this study, we report on a simple, adaptable, and non-invasive methodological approach based on ATR-FT-MIR spectroscopy and chemometrics for rapid quantification of residual phytate content in rye bran. The calibration data set exhibited a lower *RMSECV* (2.15 mM) compared to the *RMSEP* (6.32 mM), demonstrating the model´s reliability, indicating a high level of certainty. Best predictions were achieved with two factors, although using four factors resulted in the lowest *RMSECV*, emphasizing the risk of overfitting with excessive factors. The complete methodological approach displayed an *RMSEP* of 81 mg_P_∙100 g_bran_^−1^, showcasing high accuracy compared to wet chemical methods. A novel aspect of this study involved considering water absorption by bran particles, a crucial factor drastically enhancing result accuracy. Indeed, further validation and refinement are possible. However, the initial validation conducted substantiates the proof of concept, emphasizing the method´s potential.

Since ATR-FT-MIR is merely used to analyze the P content in the liquid phase, the method is transferable to other plant materials containing phytate. In addition, the method can be adapted to other real-time analytical methods such as Near-Infrared, Nuclear Magnetic Resonance or Raman spectroscopy. Since this method can be used in a scalable manner, it holds the potential for inline process control in feed material conditioning prior to feeding, even on a larger scale.

This study also demonstrates that in feeds with high intrinsic phytase activity, a large proportion of the phytate contained is initially degraded within the first hour of wet-treatment. Thus, after 1 h, wet-treatment is already more effective than the addition of exogenous phytase to animal feed rations. In addition, further insight into the behavior of phytate degradation by wet-treatment is provided and strongly suggests mass transfer limitation during the treatment. Therefore, the application of additional enzymes, e.g. xylanases or cellulases, to open the cellular matrix and thus enhance the mass transfer might be suitable to further reduce wet-treatment times.

Regardless of whether exogenous enzymes are added or intrinsically activated, e.g., by wet-treatment, feed ingredient processing prior to feeding helps to pre-digest InsP_6_ contained in the feed with maximum efficiency. Nevertheless, further research is essential to comprehend and implement feed pre-processing techniques, aiming to enhance nutrient digestibility, adjusting dietary profiles, and efficiently recycle excess P that would otherwise be diluted into the environment.

## Data Availability

The datasets generated during and/or analyzed during the current study are available from the corresponding author upon reasonable request.

## List of symbols

ATR-FT-MIR: Attenuated total reflection mid-infrared spectroscopy
*c*_*measured*_: Feedback for mass balance for P–O and P=O species, Eq. (7)
*d*_*2*__*,3*_: Sauter mean diameter
Inositol-P: Phosphorus bound to inositol (inositol-phosphorus)
InsP_1_: Inositol-monophosphate
InsP_3_: Inositol-triphosphate
InsP_4_: Inositol-tetraphosphate
InsP_5_: Inositol-pentaphosphate
InsP_6_: Inositol-hexaphosphate
*m*_*bran*_: Mass of bran
${m}_{InsP,bran}\left({t}_{0}\right)$: Amount of inositol-P present in starting material
${m}_{InsP,bran}\left(t\right)$: Amount of inositol-P present at time point t
${m}_{P,liquid phase}(t)$: Amount of P in liquid phase at time point t
*m*_*P*_: Mass of phosphorus
n: Total number of samples
Q_3_: Cumulative and volume specific particle size distribution
*R*^*2*^: Coefficient of determination
*RMSECV*: Root mean square error of cross validation
*RMSEP*: Root mean square error of prediction
rpm: Rounds per minute
*S*_*V*_: Volume-specific surface area
U: Unit [µmol·min^−1^]
*V*_*2*__*,3*_: Average volume of particle applying Sauter mean diameter
*V(m*_*bran*_*)*_*water*_: Volume of liquid phase as function of bran mass
*y*: Concentration determined by reference method
*ŷ*: Concentration determined by PLSR model using MIR data
*Ψ*: Sphericity
